# Mr. Ring, on Dr. Coxe's Cases of Vaccination

**Published:** 1805-01-01

**Authors:** John Ring

**Affiliations:** New Street, Hanover Square


To the Editors of the Medical and Physical Journal.

Gentlemen,

EVERY circumstance relative to the blue pocks decribed by Dr. Coxe, convinces me that they were spurious; owing to the subject, in whom they occurred, having previously had the small-pox.

1. There

1. There was great difficulty in producing infection.—2. The progress of the disease was uncommonly rapid.—3. The blue pock has in many other instances occurred, in my own practice and in that of others, when a person who has had either the small-pox or the cow-pock, is inoculated either with vaccine or variolous matter.

My account of the greater rapidity of vaccination in the negro is not founded on my own practice; it is, however, worthy of remark, that one very striking instance here recorded by Dr. Coxe, seems to countenance this opinion.

Dr. Coxe mentions an instance, in which matter for vaccination was taken from the arm ninety-three hours after inoculation. I have often taken it still earlier; and the same has been done by Dr. Jenner.

The mistake respecting the number of troches to be made out of the mass directed in the 4th volume of this Journal, is corrected in the next volume, p. 38. The disorder for which they are prescribed, is extremely common in this kingdom, as well as in other parts of the world; far more so than I could have imagined it to be: but those who are troubled with it, generally labour to conceal their misfortune, and endure it in silence, thinking it is of no use to complain.

I once thought it was not perfectly safe for any person to put a lozenge under the tongue when going to bed, as had been the custom in some instances; but I have now some patients who practice it with impunity, and even with considerable advantage; the delicacy of their stomachs scarcely permitting them to pursue any other course.

In a former letter, dated June 4, 1804, Dr. Coxe observes, that vaccination has appeared very opportunely, in order to prevent the ravages of this wide-wasting war from being so severely felt. He laments the lukewarmness of too many medical practitioners; who, without entertaining any doubt of the efficacy of the cow-pock, submit to inoculate for the small-pox, in compliance with the solicitations of the vulgar and ignorant part of the community; and, he might have added, with the dictates of their own sordid self-interest. He thinks such conduct criminal in the highest degree; and declares, that nothing should tempt him to follow their example.

He says, "I have lately succeeded in exciting the cow-pock with a scab nine months and three weeks old; the longest period, as far as I know, in which it has proved successful.

successful. This discovery of Mr. Bryce, respecting the scab, is of vast importance; as it will enable us to preserve the infection amongst us, I hope, without difficulty.

"In one of the last Numbers of the Medical Repository, I have given an account of a very remarkable vaccine pock, as to size. It was at least an inch and half in diameter, with the perfect character of the disease; yet, not accompanied with any extraordinary symptoms."

To this valuable communication from Dr. Coxe, I beg leave to add the following interesting letters from other Correspondents.

"Sir,

"Having been disappointed by some cow-pock matter I lately received, I take the liberty to request you will send me two or three vaccinators charged with that virus.

"I have every reason to be satisfied with the security which vaccination affords against the small-pox; having repeatedly inoculated those with variolous matter, whom I had vaccinated two and three years before, without its producing any other effect, than a local inflammation on the arm.

"As I am writing to one who interests himself so much on the subject of vaccine inoculation, and who may be numbered amongst its most strenuous and able supporters, I trust I shall be excused for pressing on your attention, a practice I have used for several years as a test of constitutional affection; and which is treated of at large, in a pamphlet written on that subject by Mr. Bryce of Edinburgh, and published in 1802. I must acknowledge, I do not attribute all the failures which are said to have occurred, in the security of vaccination against the small-pox, to the virus having produced a spurious pustule, or to the irregularity of its progress; but sometimes to the circumstance of its having excited a local effect only; which has not been succeeded by a correspondent constitutional action, on which alone the security of the patient depends. The same occurrence is continually observed in the inoculation of the small-pox; for repeated experience proves, that although the pustule on the arm be accompanied by an extensive surrounding inflammation, it is not an invariable proof of the absorption of the variolous matter into the system, from the patient's having afterwards taken the small-pox by contagion; *many instances of which have fallen within my own observation.* The only circumstance, which in in that disease can be considered as a test of constitutional affection, is its being followed by a variolous eruption; unless indeed the same practice should have been adopted, which I am recommending in the cow-pox. The vaccine disease however being followed by no eruption; the febrile action being in general so extremely slight, and having nothing to characterize it; and considering the areola on the inoculated part, although a strong, yet only a presumptive evidence of security; I have, long before the publication of Mr. Bryce's Treatise, availed myself of what I consider so decisive a test of constitutional affection, that I scarcely ever omit it. About the sixth day, when the vesicle on the inoculated part is formed, or about three days before the areola may be expected to come on, I insert the point of a lancet into the vesicle; and with the lymph which exudes, I inoculate the other arm, and as many other patients as may be present in the same state of forwardness. The progress of the second inoculation is very dissimilar from the first; it proceeds more rapidly through its stages; exhibiting an areola at the same time with the vesicle on the other arm; so that upon examining them on the ninth or tenth day, they will be both found in an equal state of forwardness, the only difference being, that the second is of a more diminutive appearance. I consider this as so decisive a test of constitutional affection, and one which scarcely ever fails to take place, especially in children, that unless it does, I never venture to warrant my patient's security against variolous infection; but advise the inoculation to be repeated at a subsequent period. Having acted in this cautious manner for several years, and attending particularly to taking the virus at the earliest period possible, I have had the most satisfactory proofs of its efficacy in resisting the contagion of the small-pox.

" I hope, Sir, you will excuse the freedom I take in offering you these observations. If they are of any importance, I know no one, under whose sanction they will be more generally attended to; and if unimportant, I presume you will readily pardon an intrusion, which proceeds from an anxious desire to establish the credit of vaccination on the basis that I am convinced it merits; and which, with proper precautions, it must finally attain.

" I am, &c.

" T. Hugo."

*Crediton, Nov.* 8, 1804.

"To Mr. RING.

"Sir,

"While I admire the very commendable zeal you have exercised for the interests of humanity, with respect to the cow-pock, it affords me a very sensible pleasure to have it in my power to adduce the clearest evidence, as far as one instance can go, of the truth of your position, stated in the Medical and Physical Journal for September, 1804, p. 282; that an insusceptibility of the small-pox at one period, is no proof the party will not be liable thereto at another.

"Several years before the cow-pock was introduced, or even spoken of here, I inoculated two children of Mr. White, of the Excise, then residing at Huntly, now at Frasersburgh; one a boy, the other a girl. The latter received the infection, and had the disease in the usual way; the former resisted it. He was again inoculated with recent matter from his sister's arm, but to no purpose.

"In the year 1801, Mr. White having three other children, wished to have them vaccinated. On this occasion I stated to him, that although his son had twice resisted infection under one state of the system, he might not under another; and that he ought to have a chance with the other children. The father's reply was, that he thought him secure against all attacks; but if I was of a contrary opinion, he left it with me to determine.

"He was therefore vaccinated again, together with the three other children. These went through the regular cow-pock; but no impression was made upon the boy, beyond what the scratch of a pin would have produced; and even this speedily disappeared.

"Determined to make a last effort, I again vaccinated him with matter taken, on the seventh day, from the arm of one of his sisters; which, at length, succeeded: the symptoms were mild, and his recovery perfect.

"His age, at the time of the first inoculations, was about two years; at the second, between six and seven. He was each time in good health; and perfectly free from eruptions.

"If the above case can be of any use, to prevent parents from placing too great a confidence in what may, at best, be only a doubtful security, it is much at your service. It cannot be in better hands.

"I am, &c.

"Alex. Thompson, Surgeon, N. Britain."

*Huntly, Oct. 20, 1802.*

The following is an extract of a letter from Mr. Hector, Surgeon, of Aberdeen, dated November 14, 1804.

"The matter I received from you, on vaccinators, answered very well; and I have just now finished inoculating the second parish in this neighbourhood, in the course of the summer. In the former there were 364 vaccinated; in the latter nearly 700. My private practice has been in proportion.

"There is, besides, an institution in this town, at which the Hospital surgeons and some others attend; where, I am informed, nearly a thousand have been vaccinated this year.

"The benefit and utility of the practice to mankind will not, I believe, now be doubted by the most incredulous; and although the progress of the infection through its different stages, in the constitution, is never severely marked, it has evidently, in many instances, a beneficial effect; by carrying off the cause, be what it will, of indifferent health in children. I have often seen very tender and weakly children, of different ages, arrive at such a state of health after vaccination, that the parents themselves, although differing in opinion on other points, respecting the efficacy of the practice, have candidly acknowledged this good effect."

The preceding letters are too valuable to be suppressed. The proposition, however, of a second vaccine inoculation, appears to me too general. I am fully convinced, that every person who has the genuine and complete cow-pock, is secure from the future infection of the small-pox, as far as he can be rendered secure, either by vaccine or variolous inoculation. It may, nevertheless, be an useful hint to those who are inexperienced, and who are not well acquainted with the characteristics of the cow-pock: It may enable them to dispel any doubt that may arise, either in their own minds, or in the minds of others. On this account, I frequently practised it in the infancy of vaccination; and Dr. Jenner and others did the same.

I am, &c.

JOHN RING.

*New Street, Hanover Square.*

